# Polymorphism of mitochondrial tRNA genes associated with the number of pigs born alive

**DOI:** 10.1186/s40104-018-0299-0

**Published:** 2018-11-26

**Authors:** Dan Wang, Chao Ning, Hai Xiang, Xianrui Zheng, Minghua Kong, Tao Yin, Jianfeng Liu, Xingbo Zhao

**Affiliations:** 1 National Engineering Laboratory for Animal Breeding; Ministry of Agricultural Key Laboratory of Animal Genetics, Breeding and Reproduction; College of Animal Science and Technology, China Agricultural University, Beijing, 100193 China; 2 School of Life Science and Engineering, Foshan University, Foshan, 528225 China

**Keywords:** Mitochondrial, Number born alive, Pig, Polymorphism, Transfer RNA

## Abstract

**Background:**

Mutations in mitochondrial tRNA genes have been widely reported association with human reproductions. It is also important to explore the effect on the number of piglets born alive (NBA). Here, 1017 sows were used to investigate the association between polymorphisms in mitochondrial tRNA genes and NBA.

**Results:**

In total, 16 mutations were found in mitochondrial tRNA genes, of which 13 mutations were significantly associated with NBA (*P* < 0.05). The reproductions of mutant carriers were significantly greater than that of wild carriers by 0.989 piglets born alive/sow farrowing. To test whether the mutations altered the structure of mitochondrial tRNAs, the secondary and tertiary structures were predicted. In result, C2255T changed the secondary structure of tRNA-Val by elongating the T stem and shrinking the T loop, and C2255T and G2259A in the tRNA-Val gene, C6217T and T6219C in the tRNA-Ala gene, and T15283C in the tRNA-Glu gene altered the tertiary structure of their tRNAs, respectively by changing the folding form of the T arm, and C16487T in the tRNA-Thr gene changed the tertiary structure of mitochondrial tRNA-Thr by influencing the folding form of the acceptor arm.

**Conclusions:**

Results highlight the effect of mitochondrial tRNA genes on the number of piglets born alive, and suggest that polymorphic sites of the tRNA genes be genetic markers for selection of pig reproduction.

**Electronic supplementary material:**

The online version of this article (10.1186/s40104-018-0299-0) contains supplementary material, which is available to authorized users.

## Background

Mitochondria participate in several important cellular processes, including apoptosis, signaling, metabolic homeostasis and biosynthesis of macromolecules such as lipids and heme [1]. Beyond these functions, mitochondria are the indispensable organelle capable of synthesizing approximately 90% of cellular ATP in vertebrates. They contain a class of cytoplasmic DNA molecules, i.e., mitochondrial DNA (mtDNA). The mammal mitogenome encodes 13 polypeptides, 22 transfer RNAs (tRNAs) and 2 ribosomal RNAs (rRNAs) [2]. The 13 essential complex subunits responsible for oxidative phosphorylation (OXPHOS) are translated by the mitochondrial separate protein synthesis machinery, which uses the 22 species of mitochondrial tRNAs (mt-tRNAs) [3, 4].

In the process of gene translation, a molecule of tRNA must be bound with the appropriate amino acid, which largely depends on the structure of the tRNA. The tRNA is canonically folded into the cloverleaf secondary structure, which is characterized by four double helical regions, the acceptor (A) stem, the dihydrouridine (D) stem and loop, the anticodon (C) stem and loop, and the TφC (T) stem and loop. These stems are composed of seven, four, six, and five base pairs, respectively. Some tRNAs contain a fifth stem-loop named extra arm, which is located in the RNA sequence between the anticodon stem and the T stem [5]. It is further folded into the tertiary structure, the inverted L-shape, which is stabilized by various tertiary interactions between the D- and T-loops, and interactions of the variable region with the D-stem-loop [5]. The L-shape can amplify the effect of the two active ends: the anticodon and the acceptor stem in a simple way [6]. However, mt-tRNAs have a noncanonical secondary structure and a number of them have strong structural deviations from cytoplasmic tRNAs. Almost all tRNAs-Ser for AGY/N codons lack the D-arm, and in some nematodes, no four-armed cloverleaf-type tRNAs are present: two tRNAs-Ser without the D-arm and 20 tRNAs without the T-arm are found [7]. Therefore, mt-tRNAs are considered as “bizarre” tRNAs [4, 8].

The mt-tRNA genes are highly susceptible to point mutations, which is a primary cause of mitochondrial dysfunction and thus leads to a few of human pathologies [4]. Numbers of researches revealed that the mutations in mt-tRNA coding genes could result in the failure of metabolism [9–12], such as T4395C in tRNA-Gln, G5821A in tRNA-Cys, A7543G in tRNA-Asp, T10454C in tRNA-Arg, A14693G in tRNA-Glu, C7492T in tRNA-Ser and A3302G in tRNA-Leu. The mutation, A3302G located at the acceptor arm of tRNA-Leu, caused the dysfunction via reducing the mitochondrial copy number [10]. In particular, the mitochondrial T7719G and A7755G mutations in the tRNA-Lys gene affected litter size in Small-tailed Han sheep and Afec-Assaf flocks, respectively [13, 14]. Nevertheless, similar reports on the genetic effect of mt-tRNA genes on pig reproduction are absent. In this study, we explored the polymorphism of mt-tRNA genes, their impacts on the secondary and tertiary structures of mt-RNAs, and the correlation between them and the number of piglets born alive (NBA).

## Methods

### Animal resource

In total, 1017 sows with 2170 records on litter size were used in this study. The pigs were from commercial breeds (Duroc, Landrace and Yorkshire) at two farms (Wangzu Pig Breeding Inc. in Xingtai, Hebei Province, and Liuma Pig Breeding Inc. in Beijing, China) and from 11 maternal lineages. There were overlaps between breeds and maternal lineages. There were 3 breeds in each of the three maternal lineages (M2, M3 and M8), 2 breeds in each of the two lineages (M1 and M11), and only one breed in each of other six lineages, as detailed in Table 1. There were gene flows among the three breeds at least in the maternal lineage. To improve the efficiency of significance testing, we used the mixed population of three breeds. The pig information, including the farm, farrowing year and season, breed, parity number, service boar, were listed in the Additional file 1: Table S1. The samples were from the ear-tag or blood tissue of the pigs.

**Table 1:** The overlap between the 11 maternal lineages and the 3 breeds in the pigs

Maternal lineage	Contained individuals	Duroc	Landrace	Yorkshire
M1	341	0	6	335
M2	134	12	118	4
M3	210	166	16	28
M4	43	0	0	43
M5	71	0	0	71
M6	56	0	0	56
M7	28	28	0	0
M8	15	8	3	4
M9	3	0	0	3
M10	108	0	108	0
M11	8	7	0	1

### Polymorphism analysis

To analyze mtDNA polymorphisms, we first extracted genomic DNA using the standard phenol/chloroform method [15]. The mitochondrial DNA was PCR-amplified using 16 primer pairs described in Additional file 1: Table S2. Subsequently, PCR products were sequenced in the Sanger method. We compared the resultant data with the *Sus scrofa* complete mitochondrion sequence (GenBank Accessible No. NC_000845.1) to identify the mtDNA variants. MEGA6 [9] and DnaSP v5 [10] were used to assemble the mitogenome.

### Statistical analysis and inference

Association analyses were performed between the number of piglets born alive and polymorphic sites within the mitochondrial tRNA, rRNA and polypeptide coding genes, and the D-loop region, by a linear mixed model approach. For each SNP, the Wald chi-squared statistic was used to examine whether the SNP was associated with the trait. The method was similar to that of Chen et al. [13] and carried out by ASReml [16]. The number born alive (response variable) was adjusted for the pig farm, farrowing year-season, breed, parity number, and service boar. In addition, the genetic background was considered based on the pedigree data. The false discovery rate (FDR) correction method in the R project (R version 3.2.5) [17] was used to control the rate of false-positive rates. We regarded the adjusted *P* < 0.05 as statistical significance.

### Prediction of tRNA structures

To test whether the point mutation affected the mt-tRNA structure, the tRNAscan-SE 2.0 [18] was applied to predict the secondary structure with or without the remarkable mutations, under the ‘default’ search mode, with the vertebrate mitochondrial genetic code and ‘vertebrate mitochondrial’ source. The tertiary structures were sequentially predicted by RNAComposer with default parameters [19, 20].

### Phylogenetic conservation analysis

A total of 16 vertebrates’ mtDNA sequences were used in the interspecific conservation analysis. These included: *Sus scrofa* (NC_000845.1), *Sus celebensis* (NC_024860.1), *Phacochoerus africanus* (NC_008830.1), *Bos Taurus* (KF926377.1), *Bos indicus* (JN817298.1), *Bos grunniens* (KU891851.1), *Bos frontalis* (MF614103.1), *Bubalus bubalis* (KX758400.1), *Ovis Aries* (AF010406.1), *Capra hircus* (KP273589.1), *Camelus bactrianus* (NC_009628.2), *Cervus elaphus* (NC_007704.2), *Leopardus guigna* (NC_028321.1), *Canis lupus* (KT901460.1), *Homo sapiens* (NC_012920.1) and *Mus musculus* (KF937873.1). The conservation index (CI) was calculated by comparing the pig nucleotide variants with another 15 vertebrates, and defined as the percentage of species from the list of 15 vertebrates with the wild-type nucleotide at that position. Notably, the CI ≥ 70% is considered as high conservation [21].

## Results

### Polymorphic sites

To see the potential association between mtDNA and the number of piglets born alive, we screened the mtDNA variants in matrilineal relatives from the 11 families. PCR-Sanger sequencing led us to identify 232 mtDNA variants, listed in Table 2 and Additional file 1: Table S3. Among these, 16 variants located at nine mt-tRNA genes, including three polymorphic sites in each of tRNA-Phe and tRNA-Ala, two sites in each of tRNA-Val, tRNA-Leu(UAG) and tRNA-Thr, and one site in each of tRNA-Cys, tRNA-Asp, tRNA-Gly and tRNA-Glu.

**Table 2:** Mitochondrial mutations (excluding synonymous mutations) and corresponding effects on the number of piglets born alive

Gene	Nucleotide mutation^a^	Codon mutation	Amid acid substitution	Sig.^b^	Grantham score
Dloop	T109C	–	–	ns	–
	T124A	–	–	ns	–
	G131A	–	–	ns	–
	mt136: ACCA-ACA	–	–	ns	–
	C145T	–	–	ns	–
	C153T	–	–	ns	–
	A158G	–	–	ns	–
	T181C	–	–	ns	–
	T241C	–	–	ns	–
	C279T	–	–	ns	–
	A294G	–	–	ns	–
	C306T	–	–	ns	–
	C323T	–	–	ns	–
	C390T	–	–	ns	–
	T405C	–	–	ns	–
	A443G	–	–	ns	–
	C452T	–	–	ns	–
	C474T	–	–	ns	–
	A501G	–	–	ns	–
	A575G	–	–	ns	–
	T992C	–	–	ns	–
	mt1013: TC-TcttataaaacaC	–	–	ns	–
	T1089C	–	–	ns	–
	A1096G	–	–	ns	–
	T1146C	–	–	ns	–
tRNA-Phe	A1225G	–	–	*	–
	G1234A	–	–	ns	–
	T1236C	–	–	*	–
12S rRNA	T1333C	–	–	ns	–
	mt1550: GA-GaA	–	–	ns	–
	T1559C	–	–	ns	–
	C1644G	–	–	ns	–
	G1826A	–	–	ns	–
	G1910A	–	–	ns	–
	T1984C	–	–	ns	–
	C1991T	–	–	ns	–
tRNA-Val	C2255T	–	–	*	–
	G2259A	–	–	*	–
16S rRNA	C2294T	–	–	ns	–
	C2534T	–	–	ns	–
	T2679C	–	–	ns	–
	C2985T	–	–	ns	–
	A3009G	–	–	ns	–
	C3023T	–	–	ns	–
	A3287G	–	–	ns	–
	T3355C	–	–	ns	–
	C3372T	–	–	ns	–
	G3561A	–	–	ns	–
	T3794A	–	–	ns	–
*ND1*	C4658T	UCC-UuC	S-F	ns	155
	C4675T	CCA-uCA	P-S	ns	74
*ND2*	A5384C	AUG-cUG	M-L	ns	15
	T5718C	AUA-AcA	M-T	ns	81
	G5801A	GUC-aUC	V-I	ns	29
	A6074G	AUU-gUU	I-V	ns	29
	G6092A	GUC-aUC	V-I	ns	29
tRNA-Ala	C6217T	–	–	*	–
	T6219C	–	–	*	–
	G6265A	–	–	ns	–
tRNA-Cys	T6429C	–	–	*	–
tRNA-Asp	G8188A	–	–	ns	–
*ATP8*	T9077C	AuU-AcC	I-T	ns	89
	T9078C	AUu-ACc	I-T	ns	89
	T9146C	UUA-UcA	L-S	ns	145
	C9155T	CCA-CuA	P-L	ns	98
*ATP6*	T9289C	AUA-AcA	M-T	ns	81
	C9333T	CUU-uUU	L-F	ns	22
	T9474C	UAU-cAU	Y-H	ns	83
	T9526C	CUA-CcA	L-P	ns	98
	A9673G	AAC-AgC	N-S	ns	46
*COX3*	C9894T	ACU-AuU	T-I	ns	89
tRNA-Gly	T10601C	–	–	*	–
*ND3*	C10674T	CUC-uUC	L-F	ns	22
	G10737A	GCA-aCA	A-T	ns	58
	T10939C	AUC-AcC	I-T	ns	89
	G10992A	GCA-aCA	A-T	ns	58
*ND4L*	G11105A	GCG-aCG	A-T	ns	58
	A11210G	AUC-gUC	I-V	ns	29
*ND4*	A12439G	AUA-gUA	M-V	ns	21
	C12596T	ACU-AuU	T-I	ns	89
tRNA-Leu	A12879G	–	–	*	–
	C12883T	–	–	*	–
*ND5*	C12999A	UCC-UaC	S-Y	ns	144
	A13034G	AAC-gAC	N-D	ns	23
	C13526T	CUU-uUU	L-F	ns	22
	T14130C	GUA-GcA	V-A	ns	64
	C14218A	UUC-UUa	F-L	ns	22
	A14234C	AAA-cAA	K-Q	ns	53
	T14601C	AUU-AcU	I-T	ns	89
	C14628T	ACA-AuA	T-M	ns	81
	C14733T	ACA-AuA	T-M	ns	81
tRNA-Glu	T15283C	–	–	*	–
*Cytb*	G16224A	GUA-aUA	V-M	ns	21
	A16281G	AGC-gGC	S-G	ns	56
	A16443G	AUC-gUC	I-V	ns	29
tRNA-Thr	C16487T	–	–	*	–
	G16531A	–	–	*	–

^a^Mutation positions according to the pig mitochondrial sequence (GenBank Accession No.: NC_000845.1)

^b^When a set of statistical inferences were simultaneously considered, multiple comparisons were conducted by the FDR using the R project. “ns” represents “not significant”, and “*” represents “significant” at the significant level of 0.05

### Effect of mitochondrial polymorphism on NBA

Association analyses were performed to evaluate the correlation between the mtDNA variants and the number of pigs born alive. Thirteen polymorphic sites in mt-tRNA genes significantly affected NBA (*P* < 0.05), including the A1225G and T1236C mutations in the tRNA-Phe gene, the C2255T and G2259A mutations in tRNA-Val, the C6217T and T6219C mutations in tRNA-Ala, T6429C in tRNA-Cys, T10601C in tRNA-Gly, A12879G and C12883T in tRNA-Leu(UAG), T15283C in tRNA-Glu, and C16487T and G16531A in the tRNA-Thr genes. The reproductions of mutant carriers were significantly greater than that of wild carriers by 0.989 piglets born alive/sow farrowing (Table 3). Furthermore, these notable mutations were assembled into haplotypes, and then the pigs were clustered into two haplotypes, which were also significantly associated with NBA (*P* = 0.039 < 0.05). On the contrary, the association study proposed that the polymorphic sites in the D-loop region and the rRNA and polypeptide coding genes were not involved in NBA (Table 2).

**Table 3:** Effects of mutations in mt-tRNA coding genes on the number of piglets born alive

Mutation^c^	Gene	NBA (Means)^d^		Q_value^d^
		Reference	Mutation	
A1225G	tRNA-Phe	9.535^a^	10.524^b^	0.048
G1234A	tRNA-Phe	9.675	10.504	0.110
T1236C	tRNA-Phe	9.535^a^	10.524^b^	0.048
C2255T	tRNA-Val	9.535^a^	10.524^b^	0.048
G2259A	tRNA-Val	9.535^a^	10.524^b^	0.048
C6217T	tRNA-Ala	9.535^a^	10.524^b^	0.048
T6219C	tRNA-Ala	9.535^a^	10.524^b^	0.048
G6265A	tRNA-Ala	9.675	10.504	0.823
T6429C	tRNA-Cys	9.535^a^	10.524^b^	0.048
G8188A	tRNA-Asp	10.364	9.922	0.110
T10601C	tRNA-Gly	9.535^a^	10.524^b^	0.048
A12879G	tRNA-Leu	9.535^a^	10.524^b^	0.048
C12883T	tRNA-Leu	9.535^a^	10.524^b^	0.048
T15283C	tRNA-Glu	9.535^a^	10.524^b^	0.048
C16487T	tRNA-Thr	9.535^a^	10.524^b^	0.048
G16531A	tRNA-Thr	9.535^a^	10.524^b^	0.048

^c^Mutation positions according to the pig mitochondrial sequence (GenBank Accession No.: NC_000845.1)

^d^“Means” represented the arithmetic average of the number of piglets born alive (NBA). The FDR method was used to conduct multiple comparisons, resulting in the Q value. The superscript annotations ‘a’ and ‘b’ meant a significant difference between groups at the 0.05 level

### Structures and non-Watson-Crick base pairs of mt-tRNAs

**Fig. 1:**
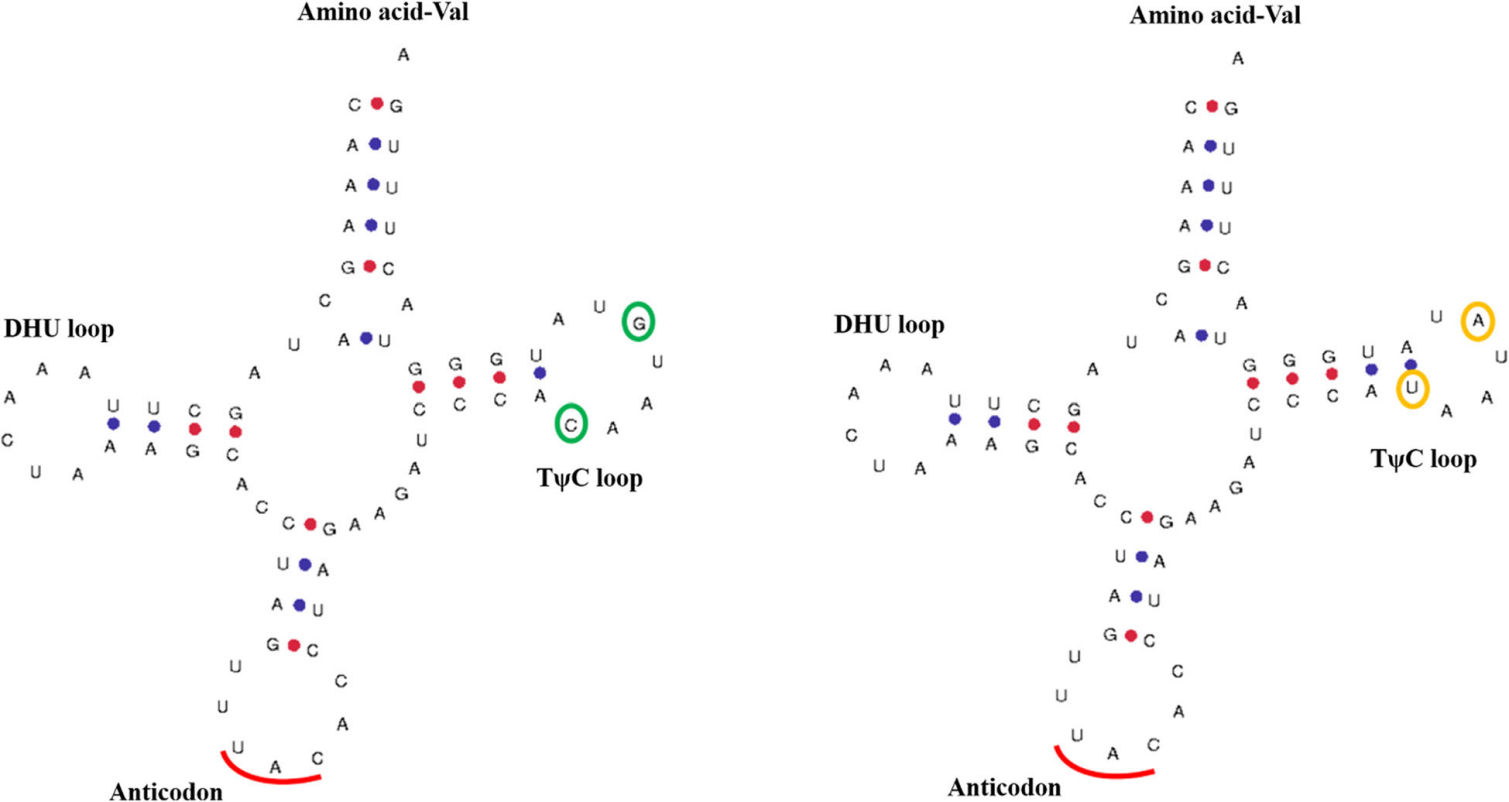
Comparison of the predicted secondary structures of mitochondrial tRNA-Val between the reference structure of NC_000845.1 (left) and the sequence with mutation sites of C2255T and G2259A (right). The circled bases were polymorphic in our study

According to the tRNAscan-SE 2.0, all of the tRNAs were folded into the cloverleaf secondary structure, displayed in Fig. 1 and Additional file 2: Figure S1. Of the 16 mutations, two sites located at the A stem of the tRNAs, four sites were in the D loop, three sites occurred in the T stem, and seven sites were situated in the T loop, detailed in Table 4. Remarkably, the mutation C2255T changed the secondary structure of tRNA-Val by elongating the T stem and shrinking the T loop.

**Table 4:** The statistics of mutation locations in mitochondrial tRNAs

TRNA location^a^	Number	tRNA gene^b^
A stem	2	tRNA-Ala(1), tRNA-Thr(1)
D loop	4	tRNA-Leu(2), tRNA-Cys(1), tRNA-Gly(1)
T loop	7	tRNA-Val(2), tRNA-Asp(1), tRNA-Thr(1), tRNA-Ala(2), tRNA-Glu(1)
T stem	3	tRNA-Phe(3)

^a^The location in the secondary structure of tRNA: D loop referred to the tRNA dihydrouridine loop; T stem and loop referred to the TφC stem and loop, respectively; A stem referred to the acceptor stem

^b^The number in the parentheses was the frequency of tRNA mutations in the corresponding genes

Fifteen non-Watson-Crick base pairs, which could be key for the three-dimensional structure of the tRNAs, were identified. Eleven of them were U-G unmatched base pairs, scatteredly distributed in the A, C, D, and T stem, and the others were A-A, A-C, C-C and C-U pairs, situated in the A stem (Tables 5 and 6).

**Table 5:** The type and frequency of non-Watson-Crick base pairs in mitochondrial tRNAs

Gene	A-A	A-C	C-C	C-U	G-U
tRNA-Phe	1				
tRNA-Val		1			
tRNA-Ala					3
tRNA-Cys					2
tRNA-Gly					1
tRNA-Glu					5
tRNA-Thr			1	1

**Table 6:** The distribution of non-Watson-Crick base pairs in mitochondrial tRNAs

Base pairs	Gene	tRNA location^a^
A-A	tRNA-Phe	A stem
A-C	tRNA-Val	A stem
C-C	tRNA-Thr	A stem
C-U	tRNA-Thr	A stem
G-U	tRNA-Ala	A stem
	tRNA-Ala	D stem
	tRNA-Ala	T stem
	tRNA-Cys	A stem
	tRNA-Cys	D stem
	tRNA-Gly	D stem
	tRNA-Glu	A stem
	tRNA-Glu	A stem
	tRNA-Glu	A stem
	tRNA-Glu	C stem
	tRNA-Glu	T stem

^a^The location in the secondary structure of tRNA: D loop referred to the tRNA dihydrouridine loop; T stem and loop referred to the TφC stem and loop, respectively; A stem referred to the acceptor stem; C stem referred to the anticodon stem

**Fig. 2:**
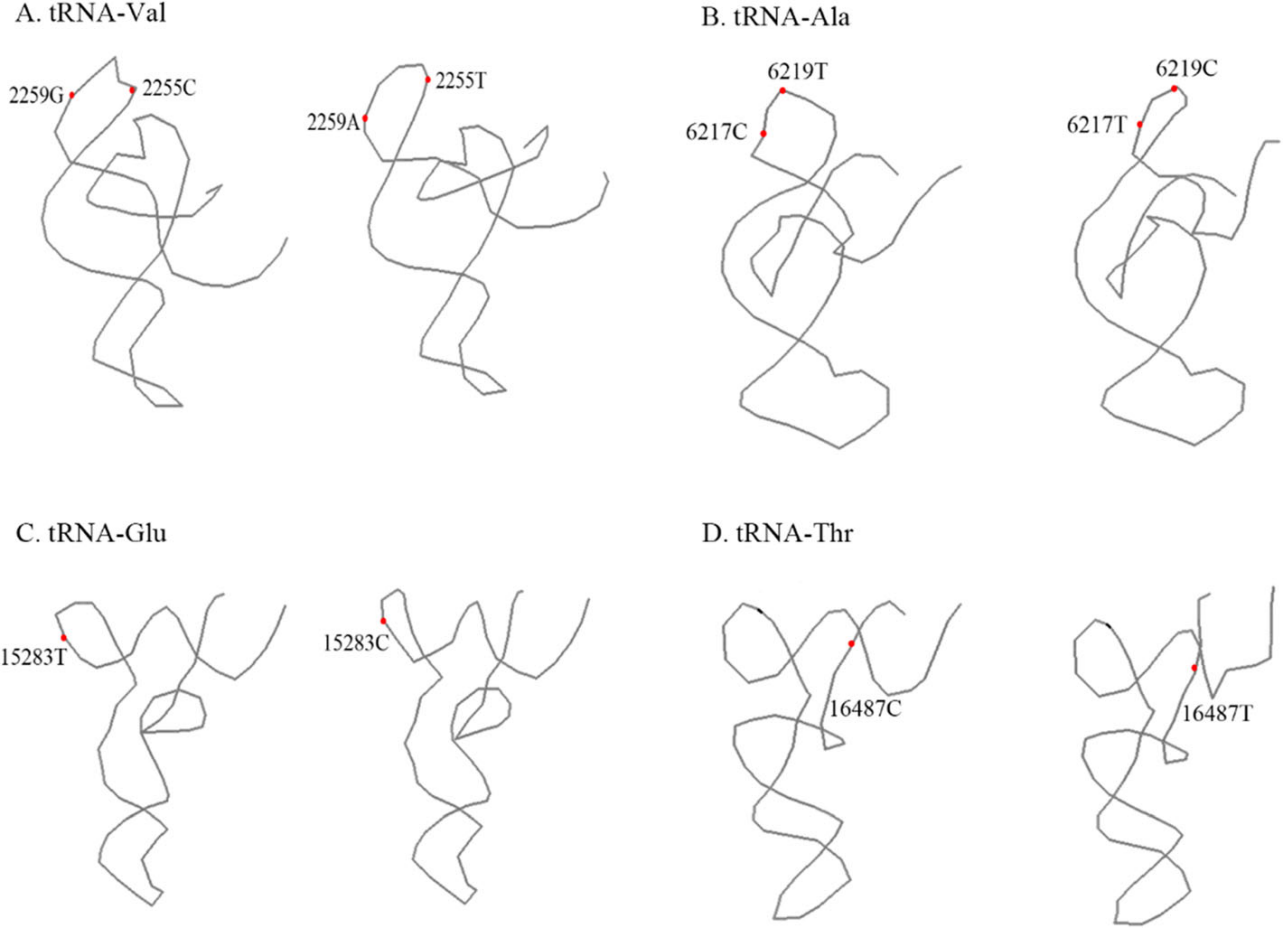
The predicted tertiary structures of mitochondrial tRNA-Val (**a**), tRNA-Ala (**b**), tRNA-Glu (**c**), and tRNA-Thr (**d**), respectively. The left ones referred to the reference structure of NC_000845.1, while the right were the structure with mutation sites. The dotted bases were polymorphic and affected the structure in the study

The tRNAs were then all folded into the L-shaped tertiary structure, showed in Fig. 2, Additional file 2: Figure S2 and Additional file 3. Notably, the C2255T and G2259A mutations affected the tertiary structure of tRNA-Val by changing the folding form of the T arm, so did C6217T and T6219C in tRNA-Ala and T15283C in tRNA-Glu. The C16487T in tRNA-Thr changed the folding form of the acceptor arm.

### Phylogenetic conservation analysis

To see whether these mt-tRNA mutations were conservative, we calculated the conservation index for each mutation. We found that the A1225G, T1236C and C6217T mutations exhibited high levels of CI (CI ≥ 70%), while the other 10 mutations had low levels of conservation (CI < 70%) (Table 7).

**Table 7:** The conservation index (CI) of each mt-tRNA mutation with significant effect on the number of born alive

Mutation	Gene	CI
A1225G	tRNA-Phe	80.00%
T1236C	tRNA-Phe	80.00%
C2255T	tRNA-Val	40.00%
G2259A	tRNA-Val	6.67%
C6217T	tRNA-Ala	86.67%
T6219C	tRNA-Ala	40.00%
T6429C	tRNA-Cys	53.33%
T10601C	tRNA-Gly	60.00%
A12879G	tRNA-Leu	26.67%
C12883T	tRNA-Leu	13.33%
T15283C	tRNA-Glu	26.67%
C16487T	tRNA-Thr	0.00%
G16531A	tRNA-Thr	20.00%

## Discussion

Mitochondrial mutations result in changes in oxidative phosphorylation enzyme complexes [22]. Since 1988, when the first pathogenic mitochondrial mutations were reported, an increasing amount of mtDNA mutations associated with a wide variety of clinical diseases have been identified [23–26]. To date, MITOMAP has featured more than 600 different pathogenic mutations in the mitochondrial genome [27]. Almost half of the mutations are located in mitochondrial tRNA coding genes, a remarkable trend, given that their sequences comprise only 10% of the mitogenome. In this study, 16 polymorphic sites were observed in 9 mt-tRNA genes, and more than 81% of them significantly affected NBA (*P* < 0.05), located in the tRNA-Phe, tRNA-Val, tRNA-Ala, tRNA-Cys, tRNA-Gly, tRNA-Leu(UAG), tRNA-Glu, and tRNA-Thr genes, respectively. The distribution of the mutations among mt-tRNA genes was widespread, concordant with that of pathogenic mutations in human mitochondria [27]. These mutations displayed a low level of conservation, 77% of them with CI lower than 70%. Transfer RNAs in animal mitochondria diverge too much in sequence among species to make comparisons and find out homologous functions from related species [28]. But several mitochondrial tRNA point mutations indeed associated with reproductive traits have been reported. The A3243G mutation in the tRNA-Leu (UUR) gene was associated with sperm motility [29]. Our study uncovered other two mutations in the tRNA-Leu(UAG) gene of great importance to reproduction. In addition, A7755G and T7719G in tRNA-Lys affected litter size in Afec-Assaf and Small-tailed Han sheep flocks, respectively [13, 14]. There were also mitochondrial genetic effects on sperm structure [30], asthenozoospermia [31], spermatozoa fertility [32] and oocyte senescence [33]. These consequences, consistent with our results, proposed the effect of mt-tRNA genes on reproduction, and suggested that tRNA polymorphic sites be genetic markers for selection on pig reproduction.

Mitogenomes are subject to a high genetic drift, while D and T arms are the places where evolutionary drift is allowed to occur [8]. In the study, base substitutions mainly occurred in D and T arms of tRNAs. All tRNAs have a global cloverleaf structure with restricted size variations, consistent with the published human mt-tRNA 2D structures [34]. The C2255T mutation changed the length of stem-loops, i.e. it elongated the T stem and shrank the T loop of tRNA-Val (Fig. 1), and made the L-shaped tertiary structure a little different, showed in Fig. 2. Additionally, the C6217T and T6219C mutations in tRNA-Ala and the T15283C mutation in tRNA-Glu affected the tertiary structure by changing the folding form of the T arm, and C16487T in tRNA-Thr changed the folding form of the acceptor arm (Fig. 2). A molecule of tRNA must be bound with the appropriate amino acid by aminoacyl-tRNA synthetase (aaRS) before translation occurs, which is related to ATP and the structure of tRNA [35]. The discovery of diseases correlated with mt-tRNA mutations provided that the effect could be traced to the destabilization of structure that destroys the native fold required for all aspects of function. The base substitutions were likely to have an impact on mt-tRNA conformation of the secondary and tertiary structures [36].

The other mutations in tRNAs didn’t change the structure conformation, and might influence translation by the paracodon. The paracodon is the second genetic code located on tRNAs identified by aaRS during translation phase of protein synthesis [37]. It is a simple structural feature, but a major determinant for establishing the identity of this tRNA [38]. The accuracy of a translation process depends on two successive independent matchings: amino acids matching with tRNAs; charged tRNAs matching with ribosome-linked mRNA. The latter is direct interaction between the anticodon and the codon [37]. The anticodon is the sequence of three adjacent nucleotides in tRNA binding to the corresponding codon and designating a specific amino acid during protein synthesis, and its mutation will be fatal, thus few changes can be preserved through evolution. However, the former depends mainly on the paracodon, which is critical. Each aaRS has a binding site for an amino acid, and another for the tRNA specific for that amino acid. Transfer RNA acts as a kind of link between the information encoded in the mRNA and the amino acid. The paracodon could not include the anticodon but may be as little as a single base pair in several instances [37, 39, 40]. Only a few paracodons have been identified for some species. Alanine specificity depended on G3 U70 in the acceptor stem of tRNA-Ala for the *Escherichia coli* [41]. tRNA-Leu could be converted to tRNA-Ser by 12 nucleotide replacements, not involving the anticodon [42]. Nucleotides in the T stem may contribute to the structure and stability of functional tRNAs [4].

In addition, the mutations in mt-tRNAs might also affect the chemical modification. RNA modifications are a regulatory layer on top of the primary RNA sequence [43], which are particularly enriched in tRNAs and critical for all aspects of tRNA functions, including folding, stability, and decoding. It was reported that loss of chemical modifications could reduce protein production or translational accuracy. It was often linked to human diseases, ranging from metabolic defects, neurological disorders to cancer [44, 45]. There are two mitochondrial-linked diseases associated with aberrant mt-tRNA modifications: mitochondrial myopathy, encephalopathy, lactic acidosis, and stroke-like episodes (MELAS) and myoclonus epilepsy associated with ragged-red fibers (MERRF) [46–48]. Nucleotide substitutions in mitochondrial tRNA genes have a large probability to obstruct the chemical modification of mt-tRNAs and thus explain the litter size effect.

No substitution occurred in the anticodon nucleotides, and the polymorphism in polypeptide coding genes had little effect on NBA. It’s reasonable because one mutation may affect DNA decoding, and subsequently leads to dysfunction of cell growth. We also evaluated the Grantham score based on the chemical properties of the amino acid side-chains [49], and found some clues that more than 91% of the amino acids were similar in chemical properties before and after variation, which resulted in little impact on NBA.

## Conclusions

In conclusion, we investigated the correlation of mitochondrial DNA polymorphisms to the number of pigs born alive, and predicted the secondary and tertiary structures of mt-tRNAs. The present study uncovered the significant associations between 13 mt-tRNA polymorphisms (A1225G, T1236C, C2255T, G2259A, C6217T, T6219C, T6429C, T10601C, A12879G, C12883T, T15283C, C16487T and G16531A) and number born alive, and the genetic effect can reach 0.989 piglets, which is potentially useful for selection of pig reproduction.

## Additional files

Additional file 1: **Table S1.** Detail information of used pigs. **Table S2.** PCR primers used for amplification of pig mitogenomes. **Table S3.** Information of synonymous mutations in pig mitogenomes. (XLSX 89 kb)

Additional file 2: **Figure S1.** The predicted secondary structures of the mitochondrial tRNAs. (A) Comparison of tRNA-Phe secondary structures between the reference structure of NC_000845.1 (left) and the structure with mutation sites (right). The green circle referred to the reference base, while the yellow was the mutation base in our study. (B-H) Comparison of secondary structures of tRNA-Val, tRNA-Ala, tRNA-Cys, tRNA-Gly, tRNA-Leu, tRNA-Glu and tRNA-Thr, respectively. **Figure S2.** The predicted tertiary structures of the mitochondrial tRNAs. (A) Comparison of tRNA-Phe tertiary structures between the reference structure of NC_000845.1 (left) and the structure with mutation sites (right). (B-H) Comparison of tertiary structures of tRNA-Val, tRNA-Ala, tRNA-Cys, tRNA-Gly, tRNA-Leu, tRNA-Glu and tRNA-Thr, respectively. (DOCX 1582 kb)

Additional file 3: The predicted tRNA 3D structures. (RAR 539 kb)

## Abbreviations

mtDNA: Mitochondrial DNA
mt-tRNA: Mitochondrial tRNA
NBA: The number of piglets born alive
