# Supplementary material for: Polymorphism of mitochondrial tRNA genes associated with the number of pigs born alive
Source: J Anim Sci Biotechnol. 2018 Nov 26;9:86. doi: 10.1186/s40104-018-0299-0 (PMC6260895; doi:10.1186/s40104-018-0299-0)
Supplement: Supplementary file 2 — Figure S1. The predicted secondary structures of the mitochondrial tRNAs. (A) Comparison of tRNA-Phe secondary structures between the reference structure of NC_000845.1 (left) and the structure with mutation sites (right). The green circle referred to the reference base, while the yellow was the mutation base in our study. (B-H) Comparison of secondary structures of tRNA-Val, tRNA-Ala, tRNA-Cys, tRNA-Gly, tRNA-Leu, tRNA-Glu and tRNA-Thr, respectively. Figure S2. The predicted tertiary structures of the mitochondrial tRNAs. (A) Comparison of tRNA-Phe tertiary structures between the reference structure of NC_000845.1 (left) and the structure with mutation sites (right). (B-H) Comparison of tertiary structures of tRNA-Val, tRNA-Ala, tRNA-Cys, tRNA-Gly, tRNA-Leu, tRNA-Glu and tRNA-Thr, respectively. (DOCX 1582 kb) [file 40104_2018_299_MOESM2_ESM.docx]

**Figure S1** The predicted secondary structures of the mitochondrial tRNAs.

A.

**
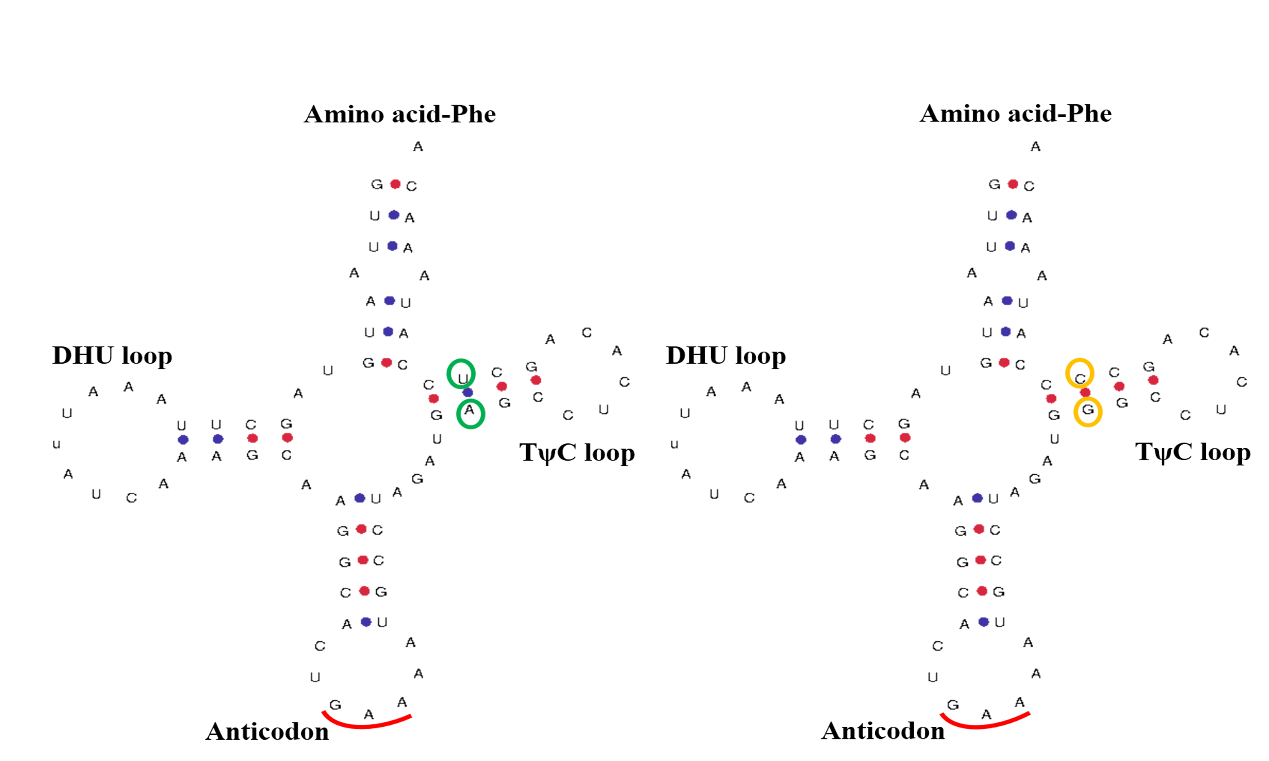
**

B.

**
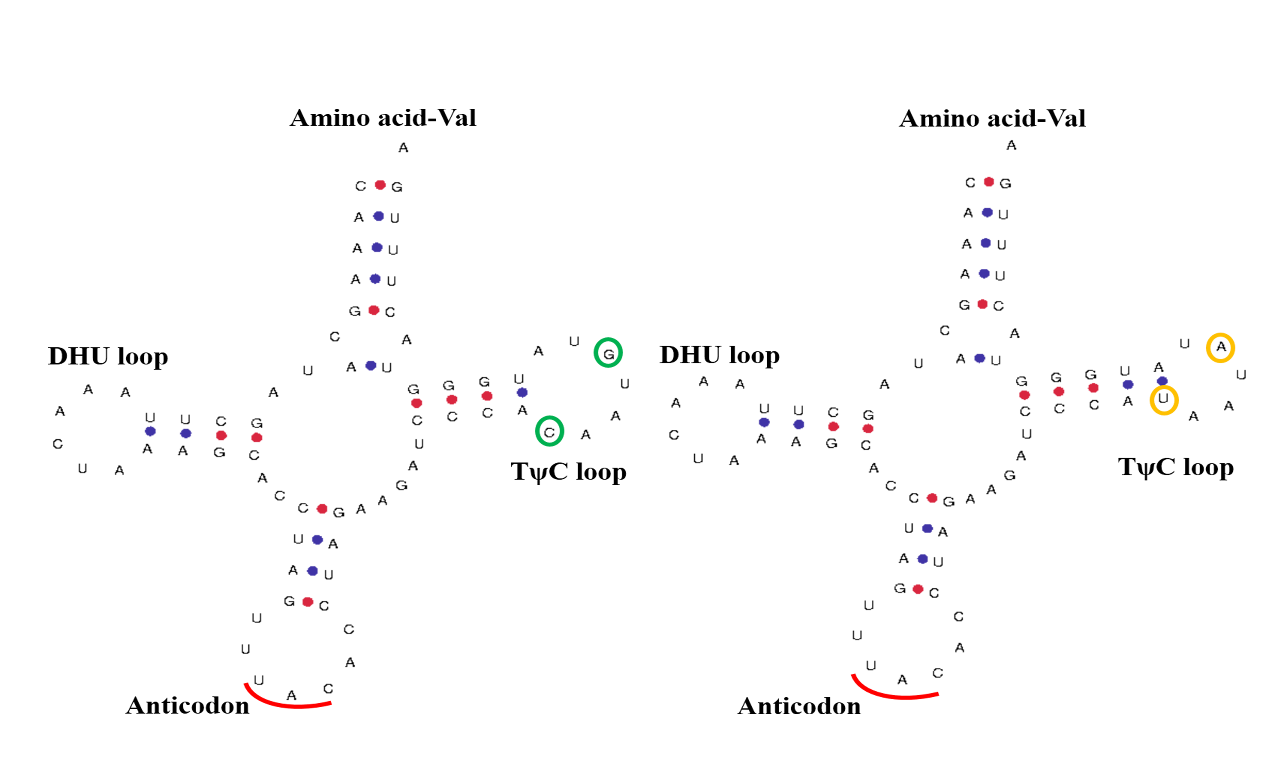
**

C.

**
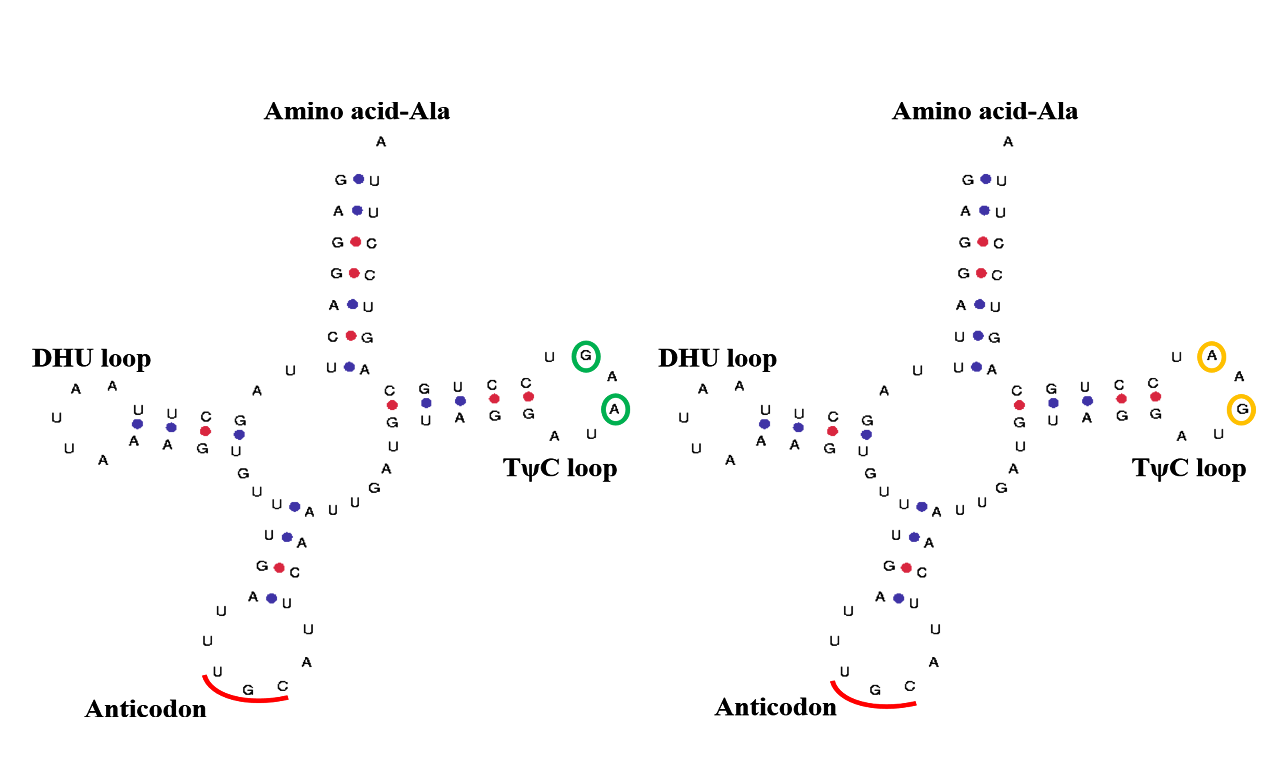
**

D.

**
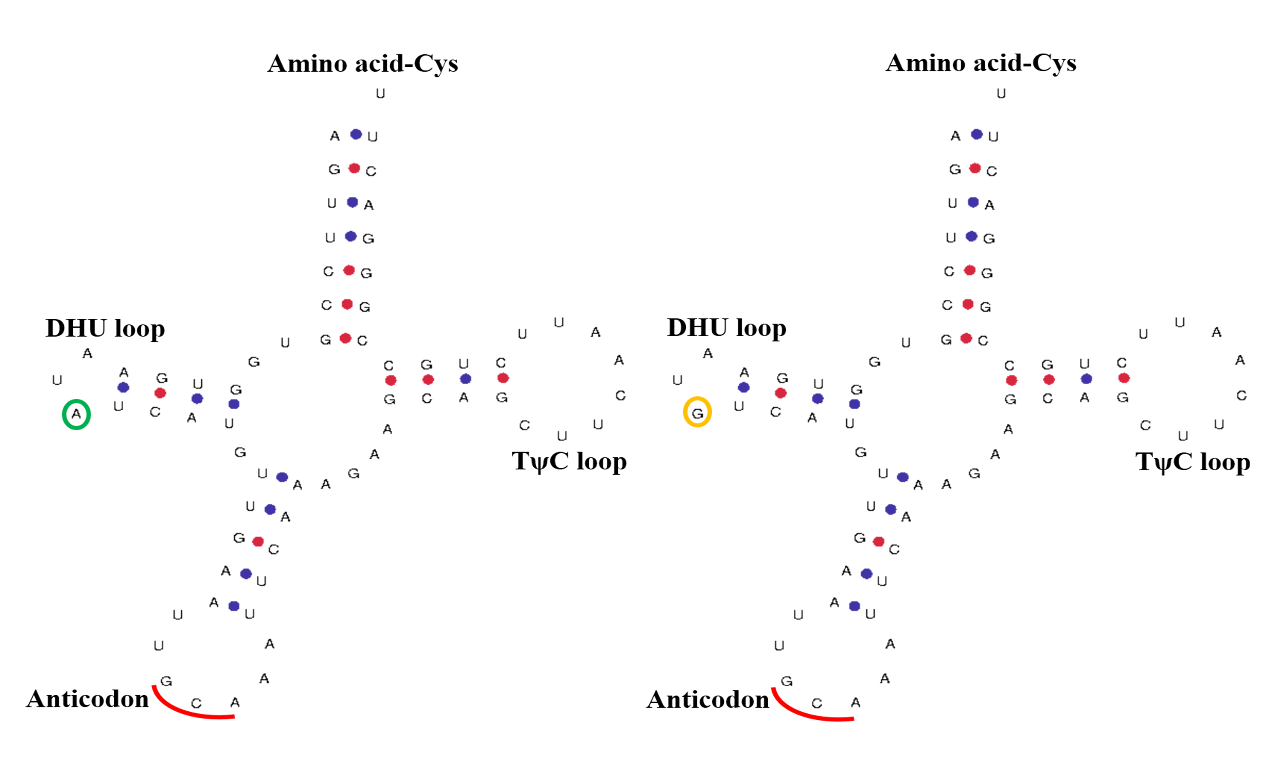
**

E.

**
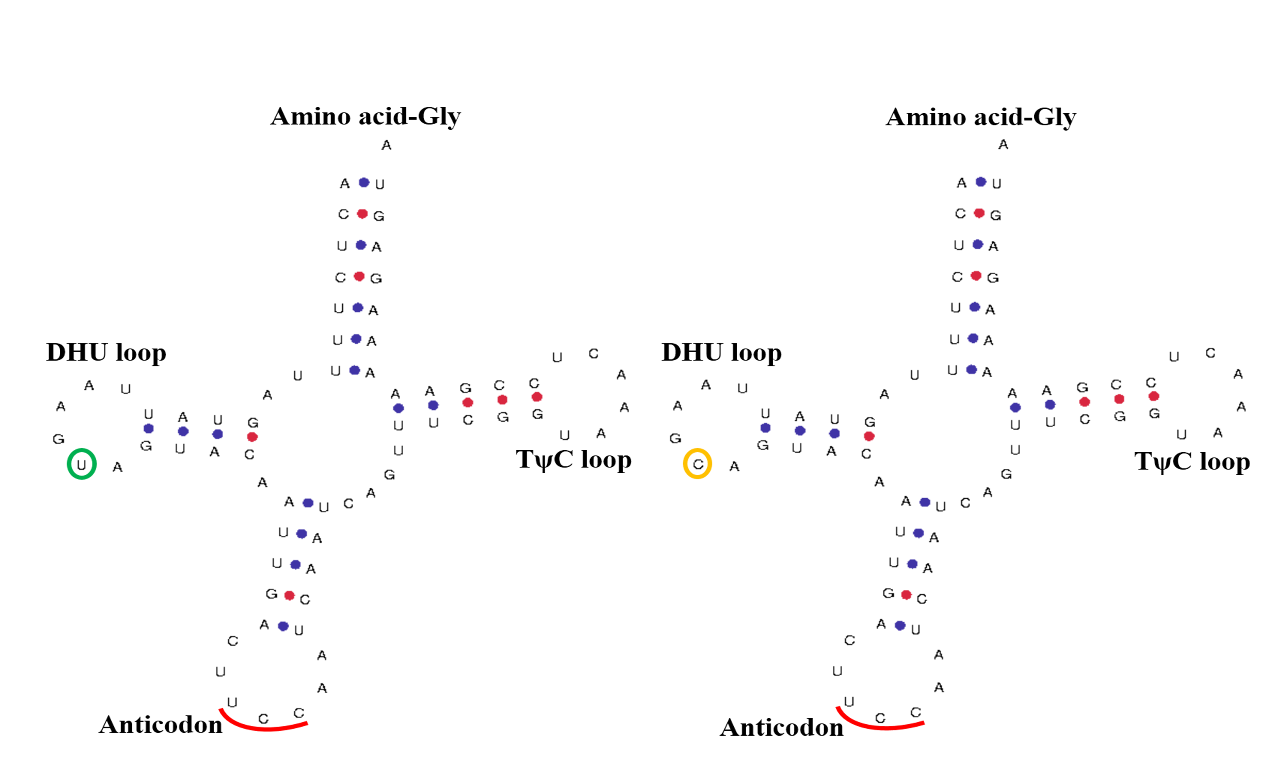
**

F.

**
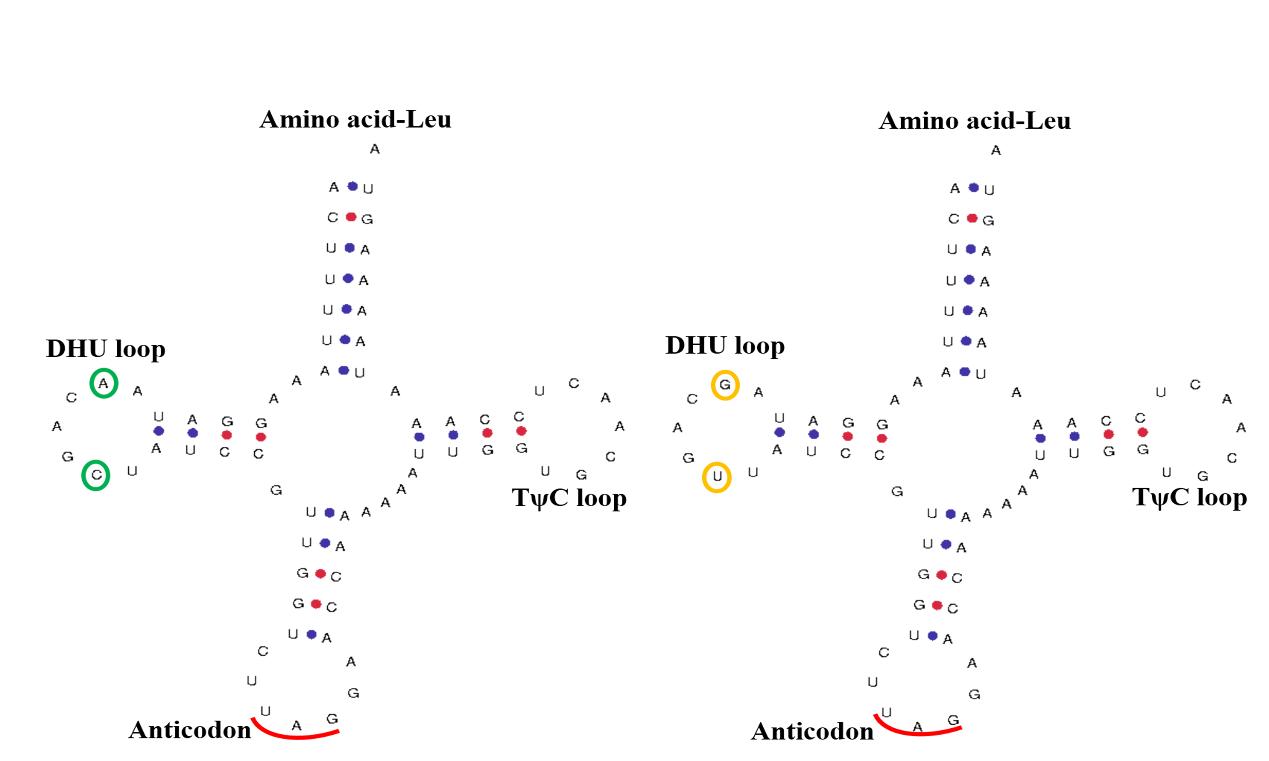
**

G.

**
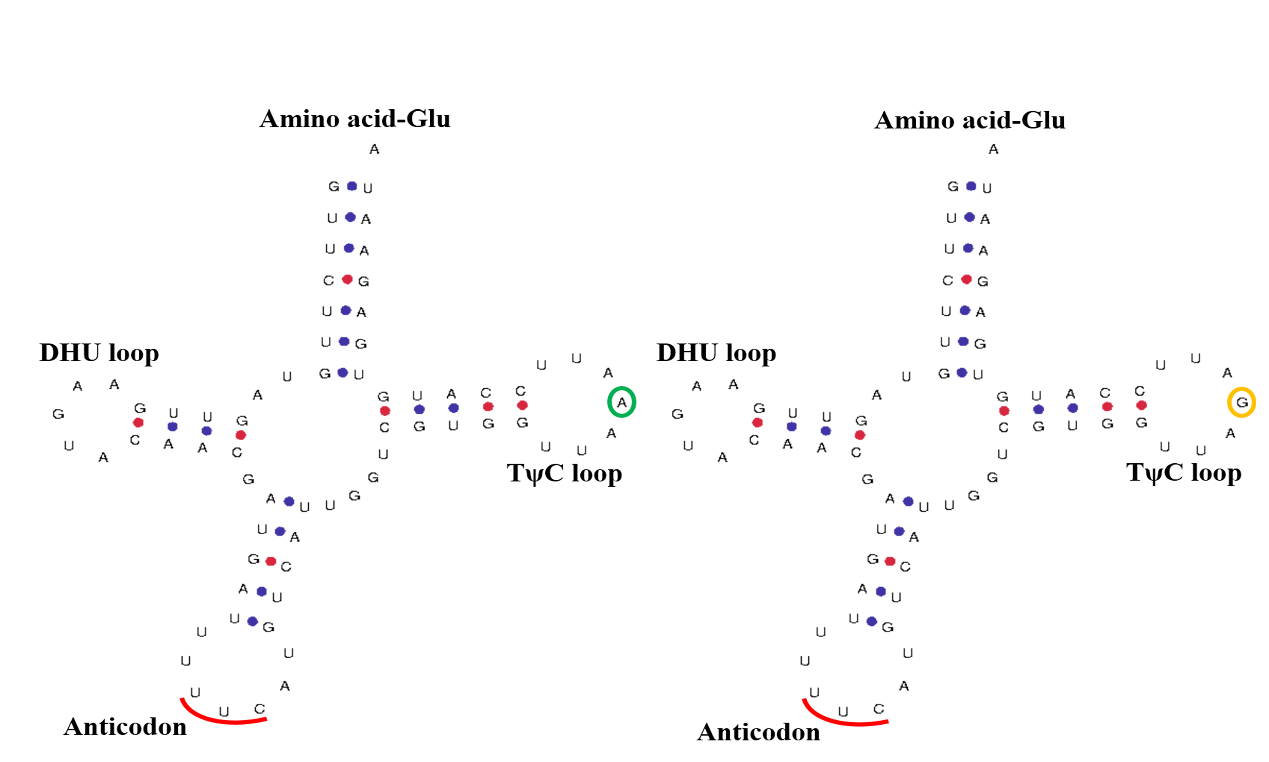
**

H.

**
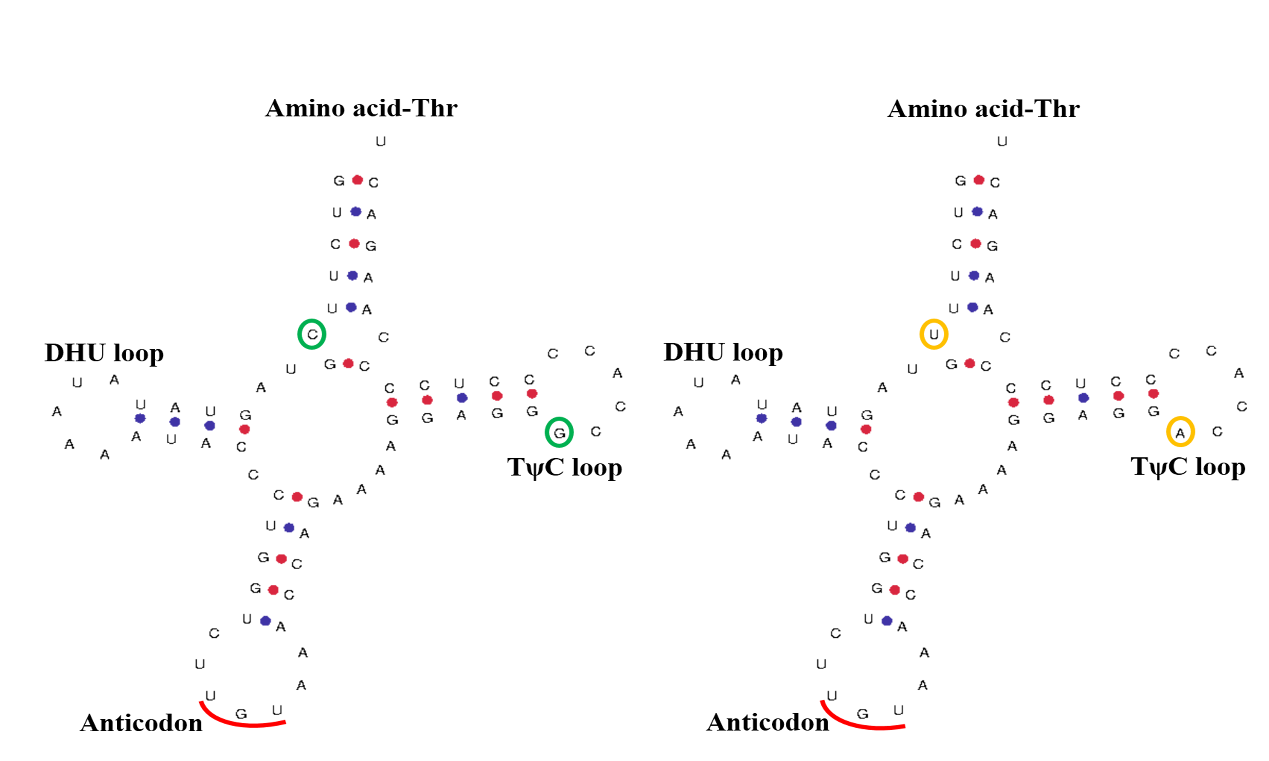
**

(A) Comparison of tRNA-Phe secondary structures between the reference structure of NC_000845.1 (left) and the structure with mutation sites (right). The green circle referred to the reference base, while the yellow was the mutation base in our study. (B-H) Comparison of secondary structures of tRNA-Val, tRNA-Ala, tRNA-Cys, tRNA-Gly, tRNA-Leu, tRNA-Glu and tRNA-Thr, respectively.

**Figure S2** The predicted tertiary structures of the mitochondrial tRNAs

A.


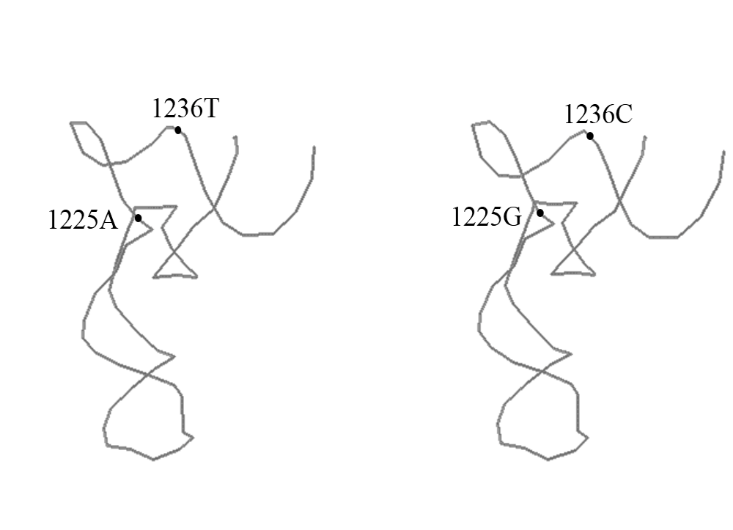


B.


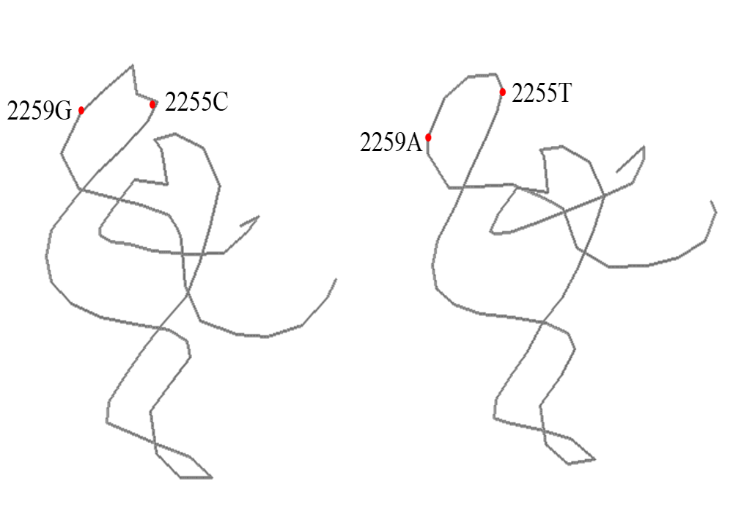


C.


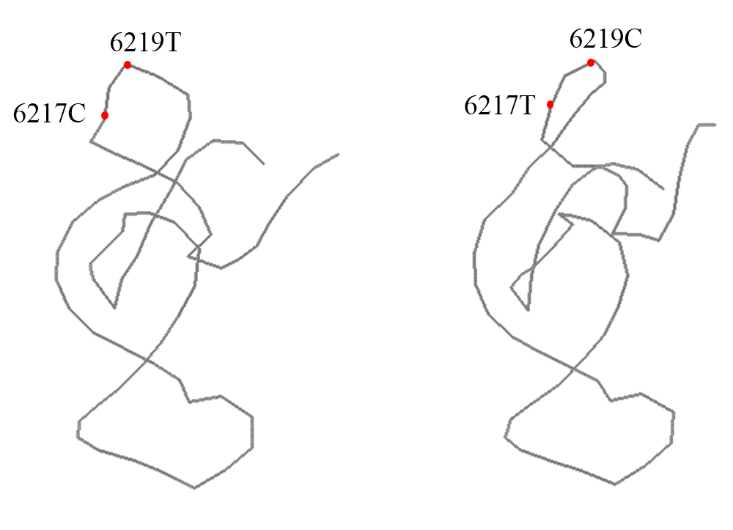


D.


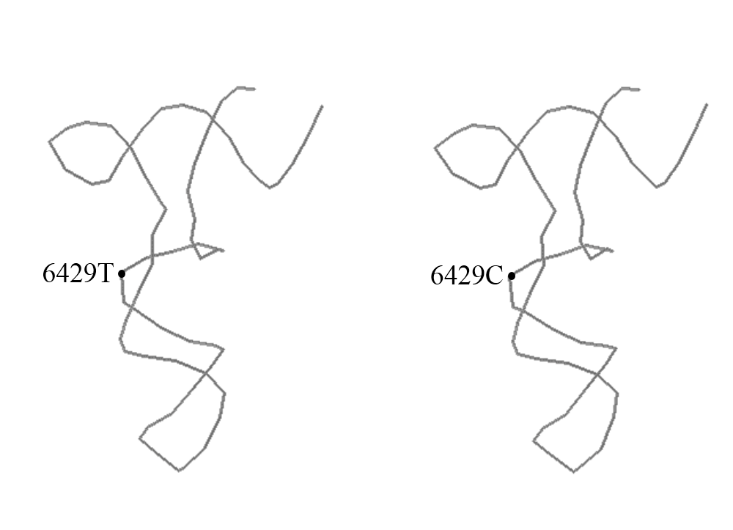


E.


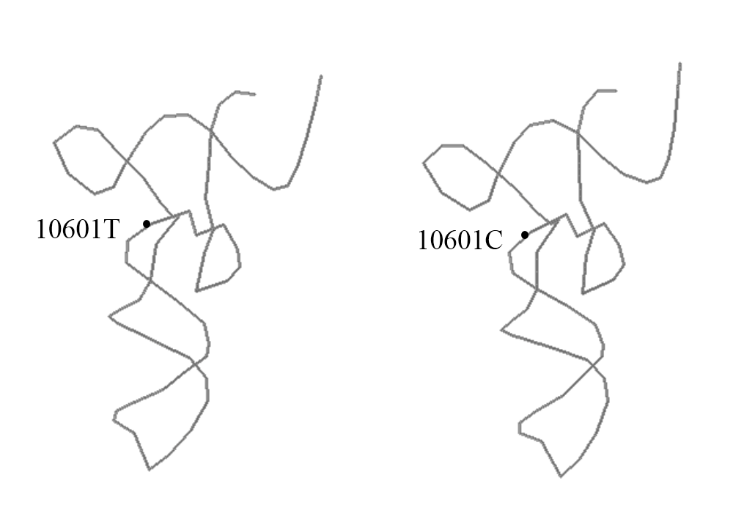


F.


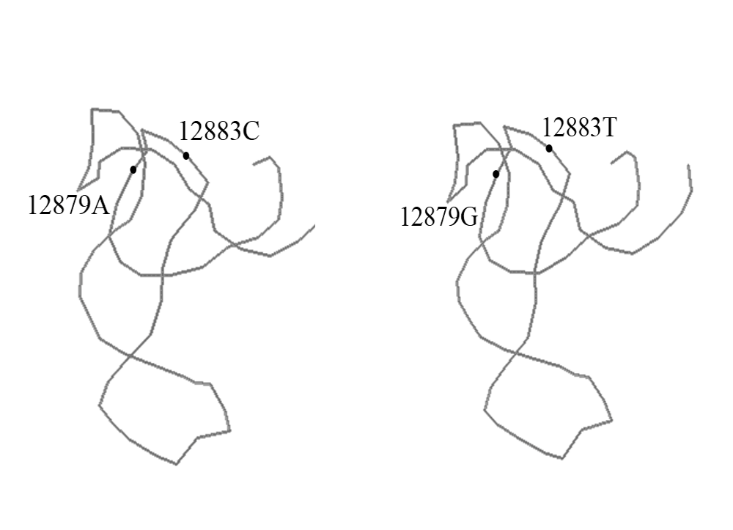


G.


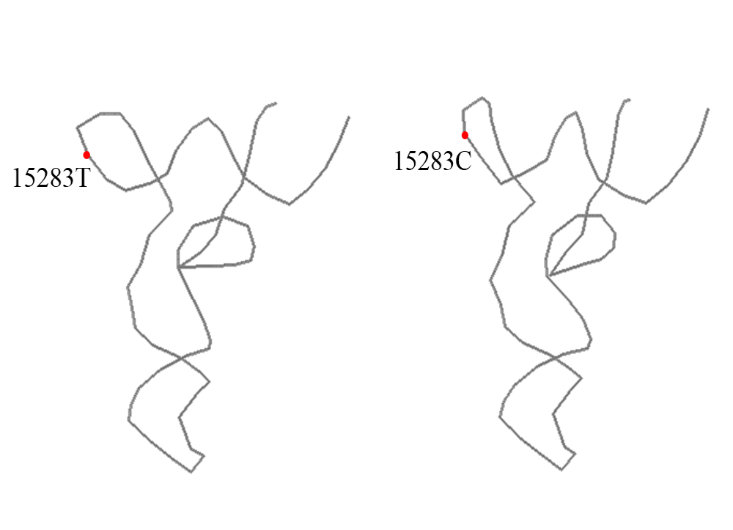


H.

**
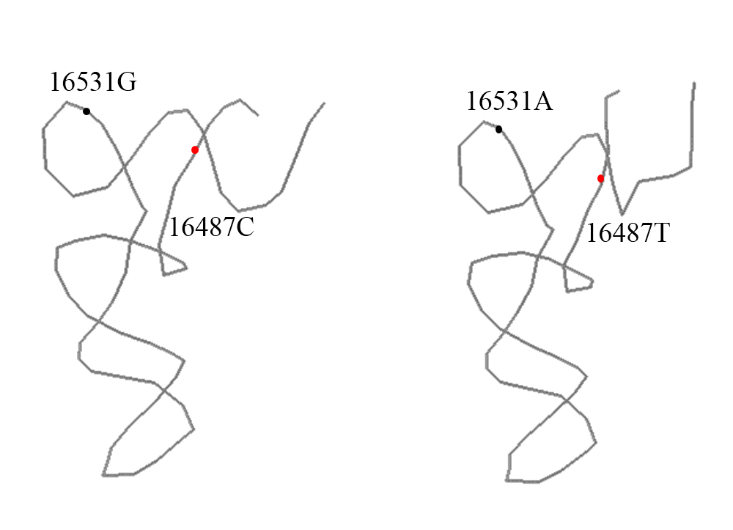
**

(A) Comparison of tRNA-Phe tertiary structures between the reference structure of NC_000845.1 (left) and the structure with mutation sites (right). (B-H) Comparison of tertiary structures of tRNA-Val, tRNA-Ala, tRNA-Cys, tRNA-Gly, tRNA-Leu, tRNA-Glu and tRNA-Thr, respectively.
